# Immunostimulatory membrane proteins potentiate *H. pylori*-induced carcinogenesis by enabling CagA translocation

**DOI:** 10.1080/19490976.2020.1862613

**Published:** 2020-12-31

**Authors:** Matthew G. Varga, Cecily R. Wood, Julia Butt, Mackenzie E. Ryan, Wei-Cheng You, Kaifeng Pan, Tim Waterboer, Meira Epplein, Carrie L. Shaffer

**Affiliations:** a Department of Epidemiology, Lineberger Comprehensive Cancer Center and Gillings School of Global Public Health, University of North Carolina, Chapel Hill, NC, USA; b Department of Veterinary Science, University of Kentucky, Lexington, KY, USA; c Infections and Cancer Epidemiology, German Cancer Research Center, Heidelberg, Germany; d Department of Microbiology, Immunology, and Molecular Genetics, University of Kentucky, Lexington, KY, USA; e Department of Cancer Epidemiology, Peking University Cancer Hospital and Institute, Beijing, China; f Department of Population Health Sciences and Duke Cancer Institute, Cancer Control and Population Sciences Program, Duke University, Durham, NC, USA; g Department of Pharmaceutical Sciences, University of Kentucky, Lexington, KY, USA

**Keywords:** *Helicobacter pylori*, gastric cancer, bacterial pathogenesis, epidemiology, CagA

## Abstract

Infection with *Helicobacter pylori* is the single greatest risk factor for developing gastric adenocarcinoma. In prospective, population-based studies, seropositivity to the uncharacterized *H. pylori* proteins Hp0305 and Hp1564 was significantly associated with cancer risk in East Asia. However, the mechanism underlying this observation has not been elucidated. Here, we show that Hp0305 and Hp1564 act in concert with previously ascribed *H. pylori* virulence mechanisms to orchestrate cellular alterations that promote gastric carcinogenesis. In samples from 546 patients exhibiting premalignant gastric lesions, seropositivity to Hp0305 and Hp1564 was significantly associated with increased gastric atrophy across all stomach conditions. *In vitro*, depletion of *Hp0305* and *Hp1564* significantly reduced levels of gastric cell-associated bacteria and markedly impaired the ability of *H. pylori* to stimulate pro-inflammatory cytokine production. Remarkably, our studies revealed that *Hp1564* is required for translocation of the oncoprotein CagA into gastric epithelial cells. Our data provide experimental insight into the molecular mechanisms governing novel *H. pylori* pathogenicity factors that are strongly associated with gastric disease and highlight the potential of Hp0305 and Hp1564 as robust molecular tools that can improve identification of individuals that are highly susceptible to gastric cancer. We demonstrate that Hp0305 and Hp1564 augment *H. pylori*-mediated inflammation and gastric cancer risk by promoting key bacteria-gastric cell interactions that facilitate delivery of oncogenic microbial cargo to target cells. Thus, therapeutically targeting microbial interactions driven by Hp0305/Hp1564 may enable focused *H. pylori* eradication strategies to prevent development of gastric malignancies in high-risk populations.

## Introduction

Infection with the gastric pathogen *Helicobacter pylori* is the single greatest risk factor for the development of gastric adenocarcinoma and is attributable for over 80% of the gastric cancer burden.^1^ Gastric adenocarcinoma is the third leading cause of cancer-related death worldwide, with over half of all incident gastric cancers occurring within East Asia.^2^ While the global prevalence of *H. pylori* exceeds 50%, less than 3% of infected individuals will develop adenocarcinoma.^1^ Therefore, due to the high incidence of this bacterium in global populations and the relatively low risk of carcinogenesis, mass eradication strategies are neither advisable nor feasible. Targeted eradication strategies in highly susceptible populations have been shown to reduce gastric cancer risk.^3,4^ Thus, identifying specific predictive factors could enable the development of powerful screening modalities to pinpoint high-risk populations that would benefit from targeted eradication and screening strategies.

The *H. pylori cag* pathogenicity island (*cag* PAI) is a strain-specific virulence factor that augments cancer risk.^5,6^ The *cag* PAI encodes a type IV secretion system (*cag* T4SS) that facilitates the delivery of peptidoglycan, nucleic acids, lipid metabolites, and the oncoprotein CagA into host cells.^7–9^ The majority of people infected by *H. pylori* harboring the *cag* T4SS do not develop cancer,^1,10,11^ suggesting that other bacterial constituents affect disease risk. Previously, we employed a serologic *H. pylori* biomarker panel to demonstrate that seropositivity to two uncharacterized *H. pylori* proteins encoded by *Hp0305* and *Hp1564* were associated with a fourfold increase in the odds of gastric cancer incidence in a consortium of eight prospective cohorts from East Asian populations.^12^ These findings were then validated as markers of gastric precancerous lesions in an independent study population from a high-risk region of Linqu County, China.^13^ Antibody response to the dual-positivity of Hp0305 and Hp1564 (referred to as OMP in previous studies) was superior at predicting incident gastric cancer risk as well as prevalent precancerous gastric lesions compared to detecting *H. pylori* alone or the secreted oncoprotein CagA.^12,13^ Despite the observed associations of Hp0305 and Hp1564 with cancer risk, the mechanism by which these proteins influence carcinogenesis remains unknown. Thus, we sought to elucidate the role of these proteins in *H. pylori*-mediated carcinogenesis in an effort to delineate promising bacterial factors that could be therapeutically targeted in novel precise intervention strategies.

## Materials and methods

### Linqu County study population

Serum samples analyzed for antibody responses to Hp0305 and Hp1564 as well as levels of pepsinogen I and II were obtained from the Linqu County intervention trial as described previously.^13,14^ Briefly, in 2002, an intervention trial was established in Linqu County, Shandong Province, China, to compare the effect of *H. pylori* treatment and selective COX-2 inhibitors on precancerous gastric lesions. In total 2,813 residents aged 35–64 were recruited from 12 randomly selected villages in Linqu County. At baseline entry into the study, participants completed a standard structured questionnaire and provided a blood sample. Participants were also screened by upper endoscopy^15^ for presence of superficial gastritis (SG), chronic atrophic gastritis (CAG), intestinal metaplasia (IM), indefinite dysplasia (Ind DYS), dysplasia (DYS), and cancer, following the criteria of the Updated Sydney System^15^ and the Padova International Classification.^16^ A written informed consent was obtained from each participant and the study was approved by the Institutional Review Board of Peking University Cancer Hospital (2016KT90).

For the initial study of validating *H. pylori* antibody biomarkers associated with precancerous gastric lesions, a total of 1,402 individuals screened by upper endoscopy at baseline were included.^13^ Of those individuals, valid pepsinogen I and II levels were available for a subset of participants, thus, the present study included only those 546 individuals (226 controls and 320 cases).

### Measurement of serum antibody responses to Hp0305 and Hp1564

Antibody responses to *H. pylori* hypothetical proteins Hp0305 and Hp1564 were measured as part of a larger multiplex serology panel including in total 13 *H. pylori* proteins as described previously.^13,17^ Briefly, *H. pylori* proteins were recombinantly expressed as Glutathione-S-transferase (GST)-tag fusion proteins in *Escherichia coli* BL21 and affinity-purified on glutathione-coated fluorescently labeled polystyrene beads (Luminex Corp.). A mixture of the differently labeled and antigen-loaded beads was then incubated with serum to allow binding of serum antibodies to the *H. pylori* proteins. Bound serum antibodies were detected by a biotin-labeled anti-human IgM/IgA/IgG secondary antibody and Streptavidin-R-phycoerythrin. A Luminex 200 analyzer (Luminex Corp.) then distinguished between the bead type and consequently the bound antigen as well as quantified the amount of bound serum antibody as median fluorescence intensity (MFI) of 100 beads per type measured. Antigen-specific cutoffs were determined as described in Epplein *et al*., 2018.^13^

### Detection of pepsinogen I and II in serum

Pepsinogen I and II levels in serum were determined by pepsinogen I and II enzyme-linked immunosorbent assay (ELISA) (Eagle Biosciences) according to manufacturer’s instructions and as described previously.^13^ The pepsinogen I and II concentration (ng/ml) in each sample was determined by provided assay standards to obtain a plate specific standard curve. A ratio of pepsinogen I over II was calculated for each sample for further analysis.

### Statistical analyses

As only a few individuals presented with normal gastric mucosa, study participants with superficial gastritis (SG) were chosen as the referent group. Thus, the distribution of demographic characteristics among individuals with precancerous lesions (CAG, IM, Ind Dys, and Dys) was compared to individuals with SG by Chi-squared test for categorical variables and Student’s t-test for continuous variables. The *H. pylori* seromarker of combined Hp0305 and Hp1564 status was applied as defined in Epplein *et al*., 2018^13^ as being either dual seronegative for both proteins, seropositive for either Hp0305 or Hp1564, or dual seropositive for both Hp0305 and Hp1564. The pepsinogen I/II ratio as a continuous variable was compared between the three Hp0305/Hp1564 sero-statuses by Wilcoxon rank-sum test, either in the overall study population or separately within each stomach condition group (SG, CAG, IM, Ind Dys, or Dys). A two-sided *p*-value<0.05 was considered statistically significant.

For *in vitro* assays, IL-8 production induced by *H. pylori* mutants was compared to corresponding IL-8 secretion levels induced by the WT strain by one-way ANOVA with Dunnett’s post-hoc correction for multiple comparisons. For experiments evaluating levels of adherent and internalized bacteria, enumerated colony forming units (CFUs) for each mutant were compared to the WT strain by two-tailed Mann-Whitney test. For qPCR studies, transcript levels produced by the *cagX* mutant were compared to the corresponding transcript levels produced by WT by unpaired two-tailed t-test.

### Bacterial strains and culture conditions

*Helicobacter pylori* 26695 (ATCC) and its isogenic derivatives were maintained on trypticase soy agar plates supplemented with 5% sheep blood (BD Biosciences) as previously described.^18^ Overnight cultures were grown in Brucella broth supplemented with 5% FBS at 37°C with 5% CO_2_. *E. coli* DH5α was grown on Luria-Bertani (LB) agar plates or in LB liquid medium with appropriate antibiotics overnight as needed for general cloning and plasmid propagation.

### Generation of H. pylori isogenic mutant derivatives

*H. pylori* mutants were generated as previously described.^18^ Briefly, *H. pylori* was transformed with a suicide plasmid in which the coding region of the target gene was replaced by either a kanamycin resistance cassette (*Hp0305*) or a chloramphenicol resistance cassette (*Hp1564*) and homologous flanking DNA sequences 500 base pairs (bp) up- and downstream of the target locus. Colonies resistant to kanamycin (12.5 μg/ml) or chloramphenicol (10 μg/ml) were PCR-verified to confirm insertion of the resistance cassette into the appropriate locus in the same orientation as operon transcription. Generation of the *Hp0305/Hp1564* double mutant was constructed by transformation of the *Hp1564::cat* derivative with the *Hp0305::kan* plasmid. Transformants were selected based on dual resistance to kanamycin and chloramphenicol, and gene deletion was verified by PCR targeting the insertion locus with comparison to the WT strain.

To complement mutants *in cis* at the *ureA* heterologous chromosomal locus, we used plasmids derived from pAD1.^18^ Plasmids were constructed to permit expression of C-terminal hemagglutinin (HA) epitope-tagged Hp0305 (pAD1-Cam^R^) and Hp1564 (pAD1-Kan^R^). Plasmid sequences were confirmed by sequencing, and constructs were used to transform *Hp0305* and *Hp1564* mutants, respectively. Insertion of complementation constructs into the *ureA* locus was confirmed by PCR amplification, and expression of the Hp0305-HA and Hp1564-HA epitope-tagged proteins was verified by immunoblotting using monoclonal anti-HA antibodies.

### Generation of TEM-CagA reporter strains

TEM-CagA expression strains were generated essentially as previously described.^19^ Briefly, a synthetic gene construct was generated comprising 500 bp upstream of the endogenous *cagA* promoter, the *E. coli blaM* gene (encoding the TEM‐1 β‐lactamase) lacking the N-terminal signal sequence under the control of the native *cagA* promoter, and 500 bp downstream of the *cagA* start codon. The synthetic TEM-CagA construct was transformed into the *cagA::cat-rdxA* strain,^18^ and counterselection on metronidazole (15 μg/ml) was performed to select positive clones expressing TEM-CagA. Insertion of the TEM-CagA construct into the *cagA* locus was verified by PCR and immunoblot analysis probing for TEM-CagA production (approximately 160 kDa vs 140 kDa for WT CagA). A TEM-CagA *cagE* mutant was generated as previously described^18^ via transformation with a suicide vector in which the *cagE* coding region was replaced with a kanamycin resistance cassette transcribed in the same direction as the operon. TEM-CagA *cagE* mutants were selected by resistance to kanamycin, and deletion of *cagE* was verified by PCR. To generate the TEM-CagA *Hp1564* mutant, the verified TEM-CagA strain was transformed with the *Hp1564::cat* construct and was selected on chloramphenicol as described for mutagenesis of the WT strain. Disruption of the *Hp1564* locus was verified by PCR. The corresponding Hp1564-HA complemented strain was generated by transformation of the TEM-CagA *Hp1564* mutant strain with the pAD-Hp1564-HA construct and selection on kanamycin. Integration of the Hp1564-HA expression construct was verified by PCR and immunoblot analysis probing for C-terminal HAepitope-tagged Hp1564.

### Human cell culture

AGS human gastric epithelial cells (ATCC CRL-1739) were cultured in RPMI 1640 medium supplemented with 10% fetal bovine serum (FBS), 2 mM L-glutamine, and 10 mM HEPES in the presence of 5% CO_2_ at 37°C. Primary adult human gastric epithelial cells (Cell Biologics H-6039) were propagated in human epithelial cell medium supplemented with ITS, EGF, hydrocortisone, L-glutamine, and 5% FBS (Cell Biologics H662). HEK293-hTLR9 and the corresponding parental HEK293 *tlr9-/-* reporter cell lines (InvivoGen) were grown in the presence of 5% CO_2_ in DMEM supplemented with 10% heat-inactivated FBS and 2 mM L-glutamine.

### IL-8 quantitation

Secretion of IL-8 by AGS cells co-cultured with *H. pylori* or its isogenic derivatives was determined using the human CXCL8 ELISA (R&D Systems). Briefly, AGS cells were infected with *H. pylori* or each isogenic mutant for 4.5 h at a multiplicity of infection (MOI) of 100. Cell-free supernatants were collected and analyzed as previously described.^18^ Biological replicate experiments were performed a minimum of at least three times.

### Immunoblot analysis of cag T4SS proteins

*H. pylori* and its derivatives were propagated on TSAII plates supplemented with 5% sheep blood (BD Biosciences) for 24 or 48 h, or in Brucella broth supplemented with 5% FBS for 16 hours. Bacterial cultures were normalized to an OD_600_ of 1.0 prior to lysis in 2X SDS buffer (Bio-Rad). Standardized samples were resolved by SDS-PAGE, transferred to nitrocellulose membranes (Bio-Rad), and analyzed by immunoblotting using either α-CagA or α-CagY polyclonal antisera.

### CagA translocation assays

CagA translocation into AGS cells was performed as described previously.^18^ Briefly, AGS cells were infected at an MOI of 100 in triplicate in for 6 h prior to at least three washes in 1X PBS to remove non-adherent bacteria. AGS cells were lysed in buffer containing 1% NP-40 with cOmplete™ mini EDTA-free protease inhibitor (Roche) and PhosSTOP phosphatase inhibitor (Roche). The soluble fraction was separated on a 7.5% gel (Bio-Rad) for immunoblot analysis probed by an anti-phosphotryosine antibody (α-PY99, Santa Cruz). Membranes were subsequently probed for total CagA (α-CagA, Santa Cruz), β-tubulin (Invitrogen), and *H. pylori* outer membrane protein (α-OMP, Santa Cruz). CagA translocation assays were performed at least four times per strain, and the ratio of translocated CagA (pY-CagA) to total CagA was determined by densitometry analysis for each strain in biological replicate experiments using ImageJ as previously described.^18^

### TEM-CagA translocation assays

TEM-CagA translocation was assayed as previously described.^19^ Briefly, AGS cells were infected by WT TEM-CagA or isogenic mutant strains for 2.5 h. After infection, cells were loaded with the fluorescent substrate CCF4‐AM in loading solution (LiveBLAzer‐FRET B/G loading kit, Invitrogen) supplemented with 1 mM probenecid (Sigma). For fluorescence quantification by plate reader assay, infected cells were incubated with loading solution at in the dark for 2.5 h, and fluorescence was measured with a Synergy H1 hybrid multi-mode plate reader (BioTek) using an excitation wavelength of 410 nm. Emission was detected at 450 nm (blue fluorescence) and 520 nm (green fluorescence). Levels of CagA translocation were defined as the ratio of emission at 450 nm_(sample‐blank)_ divided by the emission at 520 nm_(sample‐blank)_, where the blank wells contained only CCF4‐AM loading solution in cell culture media in the absence of AGS cells and bacteria. Relative CagA translocation is expressed as the ratio of 410_em_/520_em_, and data are expressed as the fold change over uninfected controls.

### TLR9 activation assays

HEK293 cells expressing a human TLR9 reporter construct (hTLR9) or parental Null1 cells (Invivogen) were infected in quadruplicate overnight at an MOI of 100 with *H. pylori* 26695 or isogenic mutant strains as previously described.^8^ TLR9 activation was quantified by measuring secreted embryonic alkaline phosphatase (SEAP) reporter activity in cell culture supernatants by QuantiBlue reagent (Invivogen). TLR9 activation was normalized to SEAP levels produced by infected *tlr9*-/- parental cells, and is expressed as the fold-change over mock-infected controls. A minimum of three biological replicate experiments were performed for each strain.

### Bacterial adherence and internalization assays

*H. pylori* or its isogenic derivatives were co-cultured with AGS gastric epithelial cells or HEK293-hTLR9 cells as described for IL-8 induction assays.^18^ After 4 h of infection, cell culture media was aspirated and wells were washed three times with PBS to remove non-adherent bacteria. A subset of wells used to evaluate total adherent and intracellular bacteria were replenished with fresh cell culture media. Intracellular levels of *H. pylori* were assessed by treating the remainder of the wells with cell culture media supplemented with gentamycin (100 μg/mL) and incubation for 1 h at 37°C with 5% CO_2_. Media was removed, and wells were washed three times in sterile PBS to remove residual media and gentamicin. Infected wells were mechanically lysed in PBS, and samples were serially diluted on blood agar for colony enumeration. A minimum of three biological replicate experiments were performed in triplicate for each bacterial strain.

### RNA isolation and real-time quantitative PCR (qPCR)

To assess transcript levels of target genes in response to host cell contact, adult primary gastric epithelial cells were either uninfected or co-cultured with *H. pylori* 26695 or isogenic derivatives for 4 to 6 h. Cells were washed with sterile PBS three times before total RNA isolation by the Direct-Zol Miniprep Plus kit (Zymo Research) following the manufacturer’s protocol. Total RNA was digested with Turbo DNase I (Invitrogen) prior to cDNA synthesis using the iScript cDNA synthesis kit (Bio-Rad) from 500 ng of total DNA-free RNA template. cDNA was diluted 1:20, and qPCR was performed using TaqMan MGB chemistry as previously described.^18^ To assess levels of target gene mRNA in planktonic culture, *H. pylori* 26695 and corresponding derivative strains were grown overnight in Brucella broth supplemented with 5% FBS, and RNA was prepared as described for co-culture samples. cDNA was generated from 500 ng of total bacterial DNA-free RNA template. As a control, equivalent concentrations of DNA-free RNA from each sample were analyzed by qPCR in parallel. The relative abundance of *Hp0305* and *Hp1564* transcripts produced when bacteria were co-cultured with primary gastric cells was calculated using the ΔΔCT method, with each transcript normalized to the abundance of the multiplexed *gyrB* endogenous control, and compared to normalized transcript levels produced by *H. pylori* in the absence of host cell contact.

## Results

### H. pylori Hp0305/Hp1564 seropositivity and decreased pepsinogen I/II ratios are associated with precancerous gastric lesions

**Figure 1.**
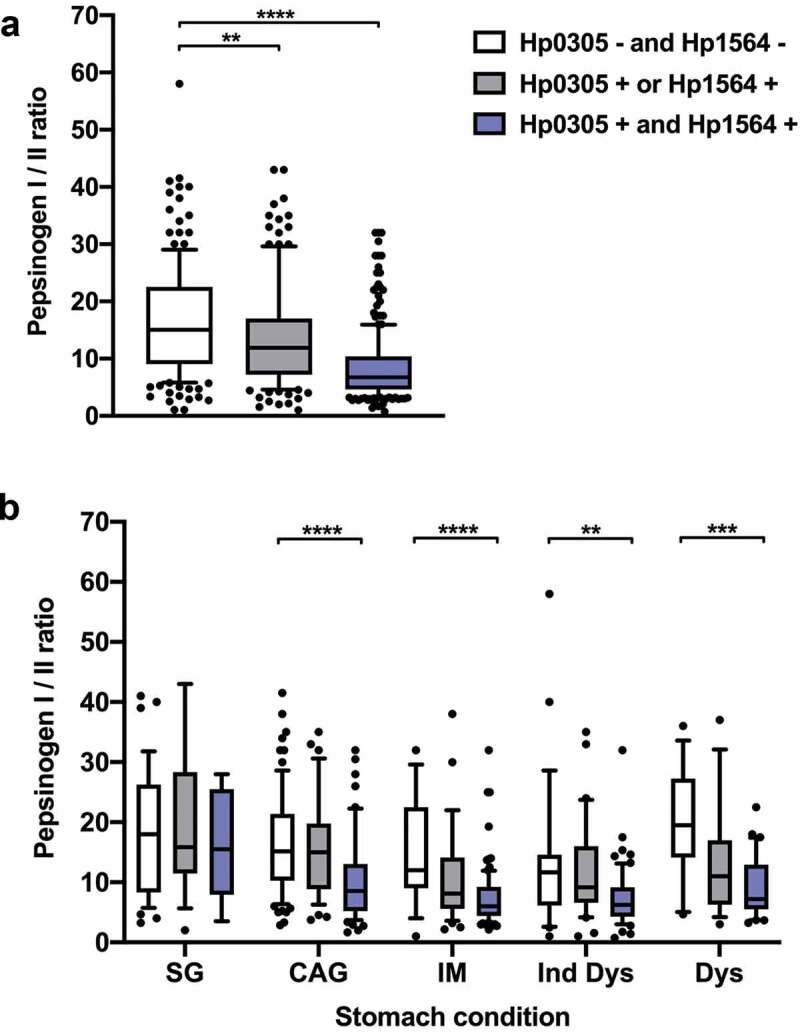
Hp0305 and Hp1564 seropositivity predict gastric inflammation

Among individuals with precancerous gastric lesions, only those with Ind Dys were more likely to be men and current smokers as compared to the reference group of individuals with SG (Table 1). However, for all precancerous lesion types the seroprevalence of *H. pylori* proteins Hp0305/Hp1564 dual-positivity was higher (CAG: 33%, IM: 65%, Ind Dys: 54%, Dys: 55%) than among individuals with SG (17%) (Table 1). A low pepsinogen I/II ratio is an established serum marker for presence of CAG in the stomach and associated with stomach cancer risk. Therefore, we assessed whether pepsinogen I/II ratios were also associated with Hp0305/Hp1564 seropositivity in our study population, and determined whether these parameters varied with type of premalignant stomach condition, i.e. SG, CAG, IM, Ind Dys, and Dys (Figure 1). Indeed, the pepsinogen I/II ratio was significantly lower in Hp0305/Hp1564 dual seropositive individuals compared to dual-negative individuals in the overall study population as well as in all precancerous lesions (CAG, IM, Ind Dys, and Dys). Thus, Hp0305/Hp1564 dual-positivity was more common among individuals with precancerous lesions compared to SG, and was concordantly associated with a lower pepsinogen I/II ratio, indicating a greater severity of gastritis among individuals with a demonstrated immune response to both *H. pylori* antigens.

**Table 1.** Demographic characteristics of the study population, Linqu County, Shandong Province, China, 2002–2004

	Referent group	Gastric precancerous lesions							
	SG (n = 60)	CAG (n = 166)	p- value*	IM (n = 142)	p- value*	Ind Dys (n = 116)	p- value*	Dys (n = 62)	p- value*
Sex, n (%)									
Female	32 (53)	98 (59)		79 (56)		43 (37)		28 (45)	
Male	28 (47)	68 (41)	0.444	63 (44)	0.764	73 (63)	**0.039**	34 (55)	0.367
Age, mean (SD)	49 (8)	49 (6)	0.582	50 (6)	0.519	50 (7)	0.602	50 (7)	0.444
Current smoker, n (%)									
No	35 (58)	113 (68)		92 (65)		47 (41)		31 (50)	
Yes	25 (42)	53 (32)	0.174	50 (35)	0.386	69 (59)	**0.025**	31 (50)	0.356
HP0305/HP1564, n (%)									
Dual-negative	37 (62)	74 (45)		15 (11)		25 (22)		12 (19)	
Either/or	13 (22)	38 (23)		34 (24)		28 (24)		16 (26)	
Dual-positive	10 (17)	54 (33)	**0.038**	93 (65)	**<0.001**	63 (54)	**<0.001**	34 (55)	**<0.001**

SG, superficial gastritis; CAG, chronic atrophic gastritis; IM, Intestinal metaplasia; Ind Dys, Indefinite Dysplasia; Dys, Dysplasia; *Compared to superficial gastritis, Chi-squared test for categorical variables, Student’s test for continuous variables, *p*-value<0.05 considered statistically significant (marked in bold font).

Pepsinogen I/II ratio by Hp0305/Hp1564 serostatus in (a) the overall study population and (b) by stomach condition. The pepsinogen I/II ratio was compared between Hp0305- and Hp1564-negative individuals (n = 165), and Hp0305- or Hp1564-positive (n = 130) as well as Hp0305 and Hp1564 dual-positive individuals (n = 251) by Wilcoxon rank-sum test (**, *p* < .01; ***, *p* < .001; ****, *p* < .0001). In (a), -, seronegative; +, seropositive; in (b), SG, superficial gastritis (n = 60); CAG, chronic atrophic gastritis (n = 166); IM, Intestinal metaplasia (n = 142); Ind Dys, Indefinite Dysplasia (n = 116); Dys, Dysplasia (n = 62).

### Hp0305 and Hp1564 influence IL-8 cytokine secretion by gastric epithelial cells

**Figure 2.**
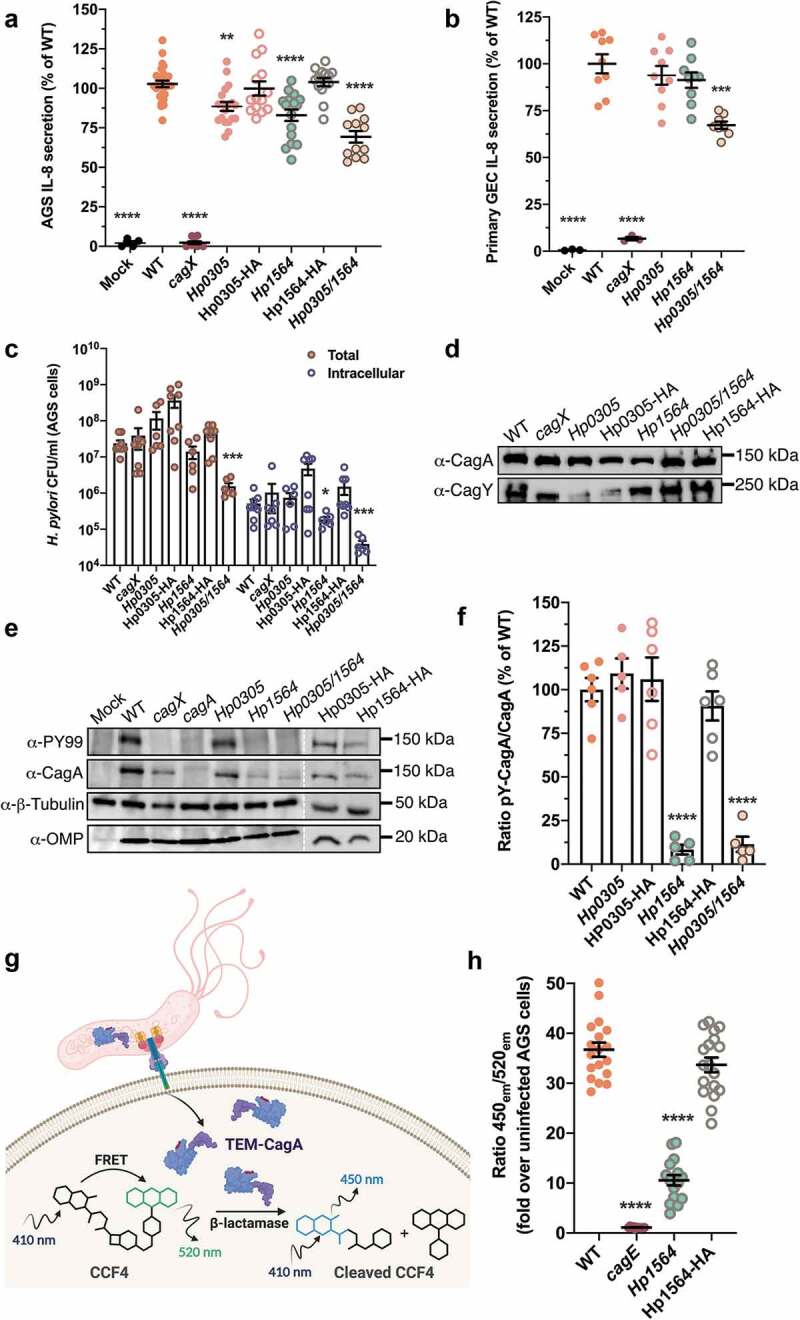
Immunostimulatory *H. pylori* proteins modulate *cag* T4SS activity

*H. pylori*-induced carcinogenesis primarily results from prolonged inflammation of the gastric mucosa. One marker of *H. pylori-*induced inflammation is elevated levels of IL-8.^20^ Therefore, we assessed the ability of strain 26695 (used to generate the multiplex serology panel employed in this study) and corresponding mutant strains lacking either *Hp0305, Hp1564*, or both *Hp0305/Hp1564* to induce *cag* T4SS-dependent IL-8 secretion in cultured, immortalized gastric cells. Compared to uninfected controls or cells infected with a *cagX* mutant strain defective in *cag* T4SS activity,^21^ we observed a significant reduction in IL-8 secretion when gastric epithelial cells were co-cultured with either *Hp0305* (*p* < .001) or *Hp1564* (*p* < .0001), an effect that was further compounded by co-culture with the double knockout *Hp0305/Hp1564* (*p* < .0001) (Figure 2(a)). Complementation of the *Hp0305* and *Hp1564* mutants with Hp0305-HA or Hp1564-HA constructs, respectively, restored IL-8 secretion to levels indistinguishable from WT (Figure 2(a)), confirming a direct role of these proteins in *cag* T4SS function. To recapitulate the gastric niche, we also co-cultured *H. pylori* strains with adult primary gastric epithelial cells (Figure 2(b)). As expected, a *cagX-*deficient mutant induced significantly less IL-8 secretion compared to the parental strain; however, only the *Hp0305/Hp1564* double mutant exhibited significant defects in eliciting IL-8 responses compared to either single mutant strain (Figure 2(b)). Together, these results suggest that Hp0305 and Hp1564 are associated with *cag* T4SS-dependent IL-8 synthesis and secretion *in vitro*, which may contribute to the increased oncogenicity observed in our population-based studies.

Levels of IL-8 induced by WT *H. pylori* and the corresponding *Hp0305, Hp1564*, and complemented mutants co-cultured with AGS gastric epithelial cells (a) and adult primary gastric epithelial cells (b) were quantified by ELISA. Data represent a minimum of three biological replicate experiments for each strain. (c) *H.pylori* were co-cultured with AGS gastric cells for 4 hours prior to gentamicin-protection assays and enumeration of the total adherent and intracellular bacterial colony forming units (CFU). (d) WT *H. pylori* and the indicated mutants were grown overnight prior to collection and cell density normalization. The levels of CagA and the *cag* T4SS structural component CagY were analyzed by immunoblot. Mutants lacking either *Hp0305* or *Hp1564* produced CagA and CagY at levels similar to the parental WT strain. (e) AGS gastric cells were co-cultured with WT *H. pylori* or the indicated mutant for 6 h. Levels of translocated tyrosine-phosphorylated CagA (α-PY99), total CagA (α-CagA), host cell cytoskeleton (α-β-tubulin), and total *H. pylori* (α-OMP) were assessed by immunoblot. (f) Semi-quantitative densitometry analysis was used to determine the levels of tyrosine phosphorylated CagA (pY-CagA) and total CagA for the indicated strains in a minimum of four biological replicate experiments. Graph depicts the ratio of translocated CagA to total CagA for each strain. (g) Schematic of quantitative TEM-CagA translocation assay. Production of the TEM‐1 β‐lactamase-CagA fusion protein (TEM-CagA) is driven by the *cagA* promoter from the endogenous locus. TEM-CagA translocation is monitored by a FRET-based assay in which CCF4 is loaded into AGS cells 2.5 hours post-infection. TEM-CagA translocation is determined by the ratio of cleaved CCF4 (emission at 450 nm) compared to intact CCF4 substrate (emission at 520 nm). (h) AGS cells were infected with WT or the indicated mutant strain and were subsequently loaded with CCF4‐AM. Turnover of CCF4 by translocated TEM‐CagA was monitored by determining the ratio of substrate emission at 450 nm to the emission at 520 nm. Ratios were normalized to cell-free controls, and are expressed as the fold change over uninfected AGS cells. Statistical significance was determined by one-way ANOVA with Dunnett’s post-hoc correction for multiple comparisons in (a), (b), (f) and (h). In (c), enumerated CFUs for each mutant were compared to the WT strain by two-tailed Mann-Whitney test. (*, *p* < .05; **, *p* < .01, ***, *p* < .001, ****, *p* < .0001 compared to WT in all panels).

### Hp1564 is required for translocation of the oncoprotein CagA into epithelial cells

As all known *cag* T4SS phenotypes require direct host cell contact, we next performed gentamicin protection assays to assess both bacterial adherence and internalization into host gastric cells. Compared to the WT strain, *Hp1564* displayed significantly reduced levels of bacterial internalization (*p* = .04), while the *Hp0305/Hp1564* double mutant displayed significantly reduced adherence and internalization (*p* < .001 for both metrics) when co-cultured with AGS gastric epithelial cells (Figure 2(c)).

*H. pylori* strains that harbor the *cag* T4SS have the capacity to translocate the bacterial oncoprotein CagA directly into gastric epithelial cells, and are associated with a higher gastric cancer risk.^11^ Therefore, we next sought to determine whether *Hp0305* and/or *Hp1564* were required for CagA delivery to gastric cells. Under standard growth conditions, deletion of *Hp0305* and/or *Hp1564* did not significantly alter levels of CagA or the *cag* T4SS structural component CagY compared to the WT or complemented strains (Figure 2(d)). However, we determined that loss of *Hp1564*, but not *Hp0305*, significantly impaired CagA translocation, an effect that was also evident in the *Hp0305/Hp1564* double mutant (Figure 2(e)). In addition, *H. pylori* lacking *Hp1564* produced less CagA when co-cultured with gastric epithelial cells compared to bacteria grown in planktonic culture (Figure 2(e)). Analysis of tyrosine-phosphorylated CagA (pY-CagA) and total CagA levels in AGS co-cultures by semi-quantitative densitometry reveled a significant decrease in the ratio of injected CagA compared to total CagA in *Hp1564*-deficient strains (Figure 2(f)). Complementation with an epitope-tagged form of Hp1564 restored the ability to translocate CagA into host cells to levels comparable to the WT strain (Figure 2(f)).

To confirm the finding that loss of *Hp1564* significantly reduces CagA translocation independent of qualitative CagA tyrosine phosphorylation assays, we established a quantitative CagA translocation system in which the *E.coli* TEM1 β-lactamase is translationally fused to the N-terminus of CagA (TEM-CagA) and expressed from the native locus driven by the endogenous *cagA* promoter.^19^ Translocation of TEM-CagA into AGS cells is monitored via TEM β-lactamase-mediated cleavage of the TEM substrate CCF4-AM^19^ and determination of the ratio of blue (cleaved CCF4-AM) to green (intact CCF4-AM) fluorescence signal (Figure 2(g)). In order to determine if *Hp1564* inactivation leads to a reduction in the levels of translocated CagA, we generated a mutant deficient in *Hp1564* and the corresponding complemented strain in the *H.pylori* 26695 [TEM-CagA] background. As anegative control, we employed an isogenic *H.pylori* 26695 [TEM-CagA]*cagE* mutant in which an ATPase required for *cag* T4SS function is inactivated. Under standard infection conditions, *H.pylori* 26695 [TEM-CagA] translocated significantly more TEM-CagA into gastric epithelial cells compared to the corresponding *cagE* and *Hp1564* isogenic mutant strains by 2.5 h of infection (Figure 2(h)). CagA translocation defects exhibited by the *H.pylori* 26695 [TEM-CagA] *Hp1564* strain were rescued by complementation with an epitope-tagged Hp1564 (Figure 2(h)), corroborating our observation that *Hp1564* mutants are associated with significantly reduced levels of tyrosine-phosphorylated CagA compared to the WT strain (Figure 2(e,f)). Taken together, these results demonstrate that *Hp1564* significantly influences CagA translocation into gastric epithelial cells.

### Hp0305 and Hp1564 are regulated in response to gastric epithelial cells

**Figure 3.**
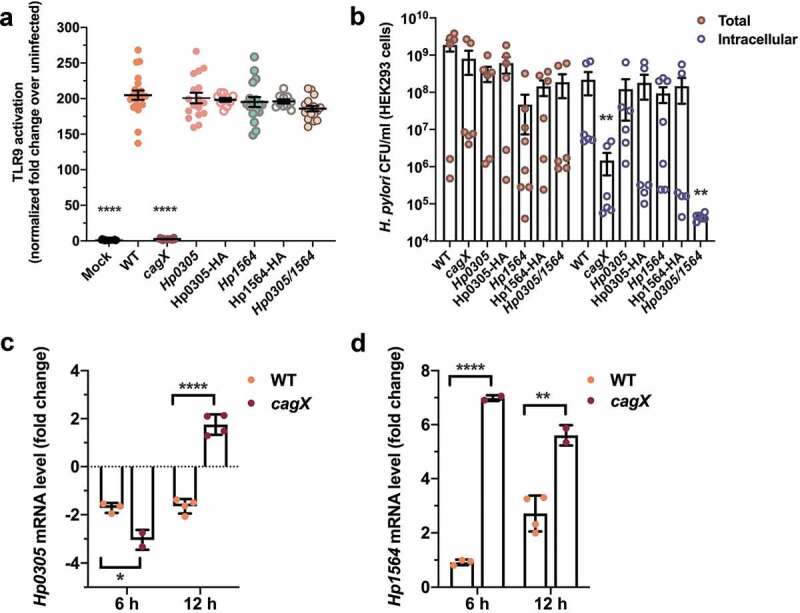
*Hp0305* and *Hp1564* are regulated in response to the gastric epithelium

In addition to the protein effector CagA, the *cag* T4SS has the capacity to deliver chromosomally-derived bacterial DNA to host cells to activate TLR9.^8^ In order to determine if Hp0305 or Hp1564 also influence *cag* T4SS-dependent TLR9 activation, a CagA translocation-independent phenotype, we employed a robust HEK293-hTLR9 reporter cell line.^8^ *H. pylori* deficient in either *Hp0305* or *Hp1564* induced high levels of TLR9 activation that were indistinguishable from the parental WT strain (Figure 3(a)). Interestingly, although TLR9 activation was not dependent on either *Hp0305* or *Hp1564, H. pylori* deficient in both *Hp0305* and *Hp1564* were internalized into HEK293-hTLR9 cells to a lesser extent than the WT strain, at levels similar to the *cagX* mutant (Figure 3(b)). These data support our previous finding that *Hp0305/1564* mutants exhibit an adherence and internalization defect when co-cultured with AGS gastric epithelial cells.

(a) WT *H. pylori* and the indicated mutant strains were co-cultured with a HEK293 reporter cell line expressing human TLR9. Normalized levels of TLR9 activation are expressed as the fold change over the corresponding mock-infected cells. (b) *H. pylori* were co-cultured HEK293-hTLR9 cells for 4 hours prior to gentamicin protection assays and enumeration of the total adherent and intracellular bacterial colony forming units (CFU). *H. pylori* were co-cultured with human adult primary gastric epithelial cells, and levels of *Hp0305* (c) or *Hp1564* (d) transcript produced by the indicated strain was assessed at 6 h and 12 h post-infection. Target transcripts were normalized to the level of *gyrB* transcript produced by each strain, and transcript abundance was compared to the level of corresponding normalized transcript produced by *H. pylori* cultured in the absence of host cell contact. In (b), enumerated CFUs for each mutant were compared to the WT strain by two-tailed Mann-Whitney test. In (c) and (d), transcript levels produced by the *cagX* mutant were compared to WT by unpaired two-tailed t-test (*, *p* < .05; **, *p* < .01, ****, *p* < .0001 compared to WT in all panels).

Lastly, to determine whether *Hp0305* and *Hp1564* are regulated in response to host cell contact, we examined the levels of *Hp0305* and *Hp1564* mRNA produced by *H. pylori* that were co-cultured with primary gastric epithelial cells. Compared to mRNA levels produced by the inoculum, contact with primary gastric epithelial cells down-regulated the production of *Hp0305* at 6- and 12-h post-infection in the WT strain (Figure 3(c)), while levels moderately increased in the *cagX* mutant strain by 12-h post-infection. In contrast, co-culture with primary gastric cells stimulated the production of *Hp1564* at 6- and 12-h post-infection (Figure 3(d)) in both strains. Compared to transcript levels observed in WT bacteria co-cultured with primary gastric epithelial cells, the expression of *Hp1564* was significantly increased in the *cagX* mutant strain under the same conditions (Figure 3(d)). These data suggest that during early infection, *H. pylori* may up-regulate the expression of *Hp1564* as a mechanism to stimulate *cag* T4SS activity. Taken together, our data demonstrate that *Hp0305* and *Hp1564* are linked to multiple *cag* T4SS-dependent phenotypes and drive key bacterial interactions with gastric cells.

## Discussion

Our previous prospective studies in East Asia have demonstrated and further validated that seroprevalence to the uncharacterized *H. pylori* proteins Hp0305 and Hp1564 is significantly associated with gastric cancer risk.^12,13^ Here, we demonstrate that seroprevalence to these proteins is also associated with increased levels of gastric atrophy (evidenced by decreased pepsinogen I/II ratios), an established hallmark of gastric inflammation.

To further define the mechanism underlying the observation that seropositivity to Hp0305 and Hp1564 is associated with gastric inflammation and gastric cancer risk, we conducted several *in vitro* studies employing *H. pylori* mutants deficient in *Hp0305, Hp1564*, or *Hp0305/Hp1564*. We demonstrate that *Hp0305* and *Hp1564* contribute, at least in part, to driving a robust inflammatory response in gastric epithelial cells. To pinpoint the role of these virulence factors in *H. pylori*-induced pathologies, we assessed whether *Hp0305* or *Hp1564* influence translocation of the oncoprotein CagA into host cells. *Hp1564*, but not *Hp0305*, is necessary for CagA translocation, while both genes are required for proper bacterial adhesion to host cells. Interestingly, TLR9 activation, a phenotype dependent on *cag* T4SS activity, was not affected by the absence of either *Hp0305* or *Hp1564*. These results suggest that loss of *Hp1564* negatively impacts CagA translocation independent of additional *cag* T4SS-associated phenotypes, including the delivery of peptidoglycan and nucleic acid substrates to host cells.

Hp0305 is an immunogenic *H. pylori* membrane protein that harbors a YceI-like domain with homology to the lipocalin fold. Previous studies have shown that Hp0305 is a component of outer membrane vesicles (OMVs) produced in response to acidic stress conditions such as those found in the stomach.^22–24^ As such, OMVs play a critical role in bacterial colonization, as well as delivery and translocation of bacterial virulence factors. Secreted OMVs are highly immuno genic, prompting production of pro-inflammatory cytokine and activation of antigen-presenting cells, as well as induction of innate and adaptive immune responses.^25^ These observations regarding *H. pylori* OMVs complement findings from the current study demonstrating the potential utility of antibody levels to Hp0305 in predicting gastric cancer risk in a cohort of East Asian individuals.^12^ Previous studies designed to characterize Hp0305 have found that purified, recombinant Hp0305 binds multiple cell types including gastric epithelial cells, macrophages, and neutrophils.^23^ Furthermore, recombinant HP0305 has been shown to induce apoptosis and TNF expression via ERK/MAPK/SFK signaling in macrophages.^23^

Similar to Hp0305, the *H. pylori* outer membrane protein Hp1564 has been repeatedly found in culture supernatants as well as the bacterial cell surface, suggesting that it is a secreted protein.^22,24^ In addition, similar to Hp0305, one study identified Hp1564 partitioned within OMVs.^26^ Other *in silico* analyses uncovered Hp1564 homology to membrane-bound methionine transporters in other bacterial species, suggesting a role of this previously uncharacterized protein in amino acid metabolism.^27^

Although the treatment regimen for *H. pylori* infection is well established, universal “test and treat” strategies are not practical given the scale of infection prevalence, the increased risk of adverse effects of *H. pylori* eradication in individuals with less-virulent strains, and adherence to antibiotic stewardship.^4,28–33^ As such, there is an urgent need to identify biomarkers that can specifically identify those patients at highest risk for gastric cancer (such as Hp0305 and Hp1564) for targeted eradication strategies. Our results strengthen previous population-based studies and delineate the mechanistic basis for their strong association with gastric cancer risk. Given the high prevalence of *H. pylori* colonization among individuals at risk for development of severe gastric disease, this work provides the framework for more efficacious treatment and prevention strategies that specifically target Hp0305/Hp1564-regulated pathways.
